# Are spliced ncRNA host genes distinct classes of lncRNAs?

**DOI:** 10.1007/s12064-020-00330-6

**Published:** 2020-11-21

**Authors:** Rituparno Sen, Jörg Fallmann, Maria Emília M. T. Walter, Peter F. Stadler

**Affiliations:** 1 Bioinformatics Group, Department of Computer Science, and Interdisciplinary Center for Bioinformatics, University of Leipzig, Härtelstraße 16-18, 04107 Leipzig, Germany; 2 Departamento de Ciência da Computação, Instituto de Ciências Exatas, Universidade de Brasília, Brasília, Brazil; 3 German Centre for Integrative Biodiversity Research (iDiv) Halle-Jena-Leipzig, Competence Center for Scalable Data Services and Solutions, and Leipzig Research Center for Civilization Diseases, University Leipzig, Leipzig, Germany; 4 Max Planck Institute for Mathematics in the Sciences, Inselstraße 22, 04103 Leipzig, Germany; 5 Institute for Theoretical Chemistry, University of Vienna, Währingerstraße 17, 1090 Wien, Austria; 6 Facultad de Ciencias, Universidad National de Colombia, Sede Bogotá, Colombia; 7 Santa Fe Institute, 1399 Hyde Park Rd., Santa Fe, NM 87501 Mexico

**Keywords:** LncRNA, Host gene, MiRNA, SnoRNA, *k*-mers, Secondary structure, Random forest, Machine learning

## Abstract

Many small nucleolar RNAs and many of the hairpin precursors of miRNAs are processed from long non-protein-coding host genes. In contrast to their highly conserved and heavily structured payload, the host genes feature poorly conserved sequences. Nevertheless, there is mounting evidence that the host genes have biological functions beyond their primary task of carrying a ncRNA as payload. So far, no connections between the function of the host genes and the function of their payloads have been reported. Here we investigate whether there is evidence for an association of host gene function or mechanisms with the type of payload. To assess this hypothesis we test whether the miRNA host genes (MIRHGs), snoRNA host genes (SNHGs), and other lncRNA host genes can be distinguished based on sequence and/or structure features unrelated to their payload. A positive answer would imply a functional and mechanistic correlation between host genes and their payload, provided the classification does not depend on the presence and type of the payload. A negative answer would indicate that to the extent that secondary functions are acquired, they are not strongly constrained by the prior, primary function of the payload. We find that the three classes can be distinguished reliably when the classifier is allowed to extract features from the payloads. They become virtually indistinguishable, however, as soon as only sequence and structure of parts of the host gene distal from the snoRNAs or miRNA payload is used for classification. This indicates that the functions of MIRHGs and SNHGs are largely independent of the functions of their payloads. Furthermore, there is no evidence that the MIRHGs and SNHGs form coherent classes of long non-coding RNAs distinguished by features other than their payloads.

**Electronic supplementary material:**

The online version of this article (10.1007/s12064-020-00330-6) contains supplementary material, which is available to authorized users.

## Introduction

A wide variety of molecular and biological functions have been reported for long non-coding RNAs (lncRNAs), recently reviewed, e.g., by Yao et al. (2019). Specific lncRNAs regulate chromosome architecture and chromatin remodeling, modulate inter- and intrachromosomal interactions, and recruit or prevent the recruitment of chromatin modifiers. Other lncRNAs regulate transcription by forming R-loops, thus recruiting transcription factors, and interfere with the Pol II machinery to inhibit transcription. Some lncRNAs are structural components of nuclear bodies. In the cytoplasm, lncRNAs regulate mRNA turnover, translation, and post-translational modification. There is, however, no clear-cut correspondence between sequence or secondary structure feature and lncRNA function. In contrast to protein-coding genes, where function is closely tied to protein families, sequence similarity is a poor predictor of functional similarity in lncRNAs. As a consequence, it has remained impossible to predict the biological function or molecular mechanism of a lncRNA from its sequence alone. This is in stark contrast also to the small, structured ncRNAs. These are readily recognized through their highly conserved sequences (such as ribosomal or spliceosomal RNAs), or by class-specific features (such as the cloverleaf shape of tRNAs or the ultra-stable hairpins of miRNAs) (Backofen et al. 2007).

Unsupervised clustering using normalized *k*-mer abundances as similarity measure revealed an association of *k*-mer profiles with lncRNA function, in particular with protein-binding properties and sub-cellular localization (Kirk et al. 2018). With a plethora of RNA-binding proteins typically recognizing a wide array of local binding motives that may be structured, modular, and gapped (Sasse et al. 2018), the correlation of short *k*-mers and function is not surprising. Nevertheless, it remains unclear whether there are distinct, well-separated classes of lncRNAs or whether the universe of lncRNAs is organized as a continuum of functions and associated molecular features.

An exception are the lncRNAs that serve as host genes for the production of small RNAs (Kapranov et al. 2007), including microRNAs (miRNAs) and small nucleolar RNAs (snoRNAs), as well as the sponges that modify the miRNA pool by acting as decoys for miRNA binding (Tay et al. 2014). Among these, the host genes of small nucleolar RNAs (snoRNAs) and the host genes of microRNAs (miRNAs) are easily recognizable because their payloads, i.e., snoRNAs or miRNAs, are evolutionarily very well conserved and thus readily identified and distinguished from each other. Due to the differences in their payloads, snoRNA host genes (SNHGs) and miRNA host genes (MIRHGs) undergo distinctive processing.

The processing of snoRNAs from SNHGs in human is linked to splicing, with snoRNAs exclusively located in introns, see, e.g., Kufel and Grzechnik (2019). In recent years, SNHGs have received rapidly growing attention in particular in cancer research, see, e.g., Goustin et al. (2019), Tamang et al. (2019), Tong et al. (2019), Yang et al. (2019), Zhao et al. (2019), Zhu et al. (2019), Zimta et al. (2020), and the references therein. SNHG15, for instance, is dysregulated in a wide variety of cancers and interacts with several distinct molecular pathways apparently using different molecular mechanisms, reviewed in Tong et al. (2019). Similar, there are several competing, not mutually exclusive, mechanistic explanations for the function of GAS5 (Goustin et al. 2019). There is strong indication that at least one of the modes of action of most SNHGs is to act as miRNA sponge (Li et al. 2020; Yang et al. 2019). The snoRNAs themselves are mostly involved in the maturation of rRNAs and snRNAs, where they direct chemical modifications, although an increasing number of secondary functions have been reported very recently (Bratkovič et al. 2020). However these do not seem to be coupled to functions of the host genes. While snoRNAs date back to a common ancestor of Eukarya and Archaea (Hoeppner and Poole 2012), non-coding SNHGs are evolutionarily much younger and seem to have appeared only comparably recently in animal evolution (Dieci et al. 2009). Biological functions of SNHGs exerted by mature, spliced SNHGs, thus, most likely have arisen secondarily.

The precursors of canonical miRNAs, whether intronic or exonic, are extracted from the primary transcript by the microprocessor complex centered around Drosha and DGCR8 (Creugny et al. 2018). A second, smaller group of miRNAs, the mirtrons, are processed with the help of the splicing machinery (Sibley et al. 2012). Functionally, miRNAs orchestrate post-transcriptional gene silencing, affecting a large fraction of the protein-coding transcriptome (Carthew and Sontheimer 2009; O’Brien et al. 2018). In contrast to SNHGs, very few MIRHGs have known functions beyond harboring their miRNA payload. Two notable exceptions are MIR100HG and MIR31HG (Sun et al. 2019). MIR100HG, for example, has been reported to interact with HuR/ELAVL1 (Sun et al. 2018) and to form RNA-DNA triplex structures with the p27 locus (Wang et al. 2018), and possibly, it may also be a miRNA sponge (ceRNA) (Huang et al. 2019b).

**Fig. 1:**
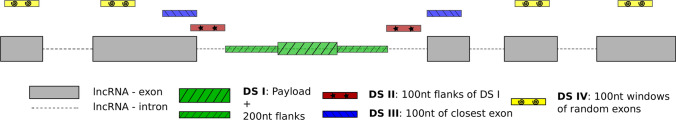
A schematic of the datasets curated for this study and their distribution over the gene body of a generic host-lncRNA. DS I (green) consists of the payload and 200nt flanking sequence. DS II (red) flanks DS-I by 100nts. DS III consists of the first 100nts of the exon closest to the annotated payload. DS IV consists of non-overlapping 100nt windows taken from random exons of the host-lncRNA. More details can be found in “Sequence retrieval and curation” section

In this contribution we ask a simple question: Are the spliced SNHGs and MIRHGs distinct classes of lncRNA? Although they do not appear as distinct clusters in the map of the lncRNA universe proposed in Kirk et al. (2018), they may be still distinguished by more complex features than *k*-mer distributions. To answer this question we use both supervised and unsupervised methods of machine learning. Specifically, we ask whether machines can be trained that reliably separate SNHGs and MIRHGs from each other and from a background set of lncRNAs that harbor neither snoRNAs nor miRNAs, which we will refer to as NoHGs.

Their payload, miRNAs and snoRNAs, has been shown to be easily distinguished by machine learning methods from each other and from other RNAs (see, e.g., Fasold et al. (2011), Saçar and Allmer (2014), Abbas et al. (2016), Georgakilas et al. (2020), and the references therein). We therefore have to ask whether the lncRNA classes remain distinguishable when the sequence information of the payload—and possibly also of regions that are involved in the processing of the payload ncRNAs—is withheld from the classifier. To this end, we devise four different collections of subsequences (see Fig. 1) and train classifiers with several different types of features (described in detail in “Materials and methods” section). As we shall see in “Results” section, the three RNA classes are only distinguishable based on sequence regions covering the payload or regions likely associated with the processing of the payload. Using sequences far away from the payload to focus on payload-independent properties, no reliable classifiers could be trained. Unsupervised clustering methods also fail to separate the three lncRNA classes (“Unsupervised clustering” section). Finally, we specifically test in “MicroRNA target sites as a feature” section  whether there is evidence for a specific function of SNHGs and/or MIRHG as sponges, again with negative results.

## Materials and methods

### Sequence retrieval and curation

For a comprehensive investigation into the separability of SNHGs, MIRHGs, and NoHGs we first had to curate data from available annotation and define sets of features than can be used reliably for training and prediction. MiRNA sequences were collected from miRBase (Kozomara et al. 2019), snoRNA sequences from the Human snoRNA Atlas (Jorjani et al. 2016), and snoDB (Bouchard-Bourelle et al. 2020), and lncRNA sequences were retrieved from GENCODE v29 and GENCODE v33 (Frankish et al. 2019), where multiple lncRNA isoforms were annotated we used the longest one as representative. The coordinates of miRNA precursors from miRBase were used to identify MIRHGs and the exact position of the payload(s) within the MIRHG. All overlaps were computed with the bedtools suite (Quinlan and Hall 2010) and customized Python scripts. Since all snoRNAs are intronic, no further processing was needed for SNHGs. In MIRHGs we defined both intronic and exonic miRNA precursors with 100 nt flanking sequence as the “payload.” The intron/exon annotation provided by the GENCODE annotation was used to define the exonic part of the lncRNA.

### Datasets

The four datasets differ in the regions of the lncRNA gene that are included, see Fig. 1 for an illustration. All data used for classification were extracted based on GENCODE annotation (version 33) using custom scripts and the bedtools suite (Quinlan and Hall 2010). For training and testing, datasets were always balanced based on the smallest number of sequences available for any given class, to prevent prediction artifacts (Table 1).

**Table 1:** Distribution of sequences contained in each dataset

	Dataset 1	Dataset 2	Dataset 3	Dataset 4
SNHGs	345	690	1287	162
MIRHGs	400	800	464	168
NoHGs	400	750	2101	750

*Dataset 1: payload included* For a set of 400 MIRHGs and 345 SNHGs, we extracted the payload sequence together with 100nt segments/fragments from both upstream and downstream flanks. Flanks are expected to contain information necessary for the processing of the payload, such as the processing of pre-miRNAs by the DROSHA/DICER machinery. As negative control set we collect 400 subsequences of random lncRNAs excluding overlaps with our positive sets on gene and sequence level. Sequence length was set to 500nt as this was the mean length of sequences in our positive sets.

*Dataset 2: flanking sequence* To investigate the influence of the information derived directly from the payload, we next prepared a dataset consisting of 800 samples from MIRHGs, 690 samples from SNHGs, and 750 samples from NoHGs. For the host genes we extracted 100nt windows flanking the regions, i.e., both upstream and downstream used as dataset 1. Overlaps between extracted sequences were discarded. These can arise from closely spaced payloads. This makes sure we have no information on the actual payload, while we preserve close-by context. Here we do not focus on the genic region of these flanks. As negative set we again derive random subsequences of lncRNAs without overlap with the positive set, this time of size 100nt to match the positive sequences.

*Dataset 3: adjacent exonic sequence* Since the genic sequence information itself is of particular interest, we constructed a set that is similar to dataset 2, with the additional constraint that 100nt sequences are chosen from the closest exon up- and downstream of the payload. Dataset comprises 464 miRNA and 1287 snoRNA host gene sequences and differs from dataset 1 by excluding payload as well as the intronic sequence surrounding the payload. As negative set we selected at random 2101 exonic regions of length 100 from randomly picked lncRNAs without overlap to the positive set.

*Dataset 4: sequences far away from the payload* To avoid any localized effect associated with the payload, we constructed dataset 4 by selecting multiple, non-overlapping regions of length 100nt from the exonic parts of the host genes. This dataset contains 168 sequences extracted from miRNA precursors and 162 extracted sequences from snoRNA precursors. For the negative set we chose multiple, non-overlapping, 100nt windows of random exons of random lncRNA genes without overlap with the positive set, amounting to 750 sequences.

### Features

The following features were computed for each of the sequences in the four datasets described above:

1. *k*-mer counts for $2\le k\le 7$ were calculated using jellyfish (Marçais and Kingsford 2011). From these data, a triple $(key, kmer\_norm, freq)$ was computed for each *k*-mer and each transcript. *key* is a categorical variable describing presence or absence of the *k*-mer in a given sequence, *freq* is its relative frequency, and *kmer_norm* is the normalized count of the *k*-mer. The entries were converted into features using a MultiLabelBinarizer class from scikit-learn  (Pedregosa et al. 2011)) as binarizer.
2. The Fickett score (Fickett 1982), a measure for the coding potential of sequences, was computed with a custom script inspired by Wang et al. (2013).
3. Sequence conservation scores were obtained from the UCSC Table Browser (PhastCons30way), and the scores for individual exons were extracted (Kent et al. 2002; Karolchik et al. 2004). The PhastCons score was used in two ways, once as mean of all the scores across all the nucleotides and as normalized scores after being divided into 5% bins.
4. RNA secondary structure is often better conserved than the sequence. We therefore utilized structural attributes of the sequences. Thus, pairing probabilities of the nucleotides were calculated using RNAplfold  (Bernhart et al. 2006). Three different windows of lengths 60, 80, 120 were used for the calculation. Each window was divided into 20 bins, binary encoding each position that falls into the bin with 1 and the rest of the positions with 0.

Since at least a fraction of the annotated lncRNAs is annotated from incomplete transcript models (Sen et al. 2017), we did not use parameters such as the number of exons, the transcript length, or polyadenylation in any of the classification tasks.

### Machine learning and evaluation framework

*Supervised machine learning* Since we are primarily interested in a three-way classification, we used random forests. A major advantage of random forest approaches is that they enable us to rank features based on their importance for classification. This allows us to investigate the latter in detail or discard less useful ones to improve training accuracy and time. We used 100 trees for the initial classification, at default settings. Tenfold and fivefold cross-validation was performed with tree sizes [100, 300, 500, 1000], both impurity criteria (Gini index and entropy), bootstrapping, and taking out of bag samples. Hyperparameters were optimized using grid search over all combinations of these parameters. For each combination of feature and dataset, we chose the best performing hyperparameter setting (details can be found in supl.tab.3). We found only small differences in prediction accuracies between training runs on the available parameter space. This is likely related to the moderate size of available datasets. All training, testing, and cross-validation were performed with Scikit-learn  (Pedregosa et al. 2011).

*Unsupervised machine learning* As an alternative approach we also tested unsupervised clustering. To this end we used the same four datasets and the same features. To obtain a visual overview of the data we used principal component analysis (PCA) on the *k*-mer vectors as implemented in R. We then used *k*-means clustering in attempt to distinguish the sequences into three distinct clusters (Hartigan and Wong 1979). We further evaluated a convolutional neural network (CNN)-based autoencoder instead of a classical clustering approach. For this purpose the *Keras* package (https://keras.io/) within the *TensorFlow* framework (Abadi et al. 2016) was applied. We calculated accuracy, F-measure, and rand index to evaluate the quality of the clusters w.r.t. distinguishing the three classes of lncRNAs.

## Results

To answer the underlying question of whether or not SNHGs and MIRHGs are indeed distinct classes of lncRNAs beyond their payloads, we ask to what extent they can be distinguished from each other and from a NoHG by means of machine learning techniques. Since it is well known that the payloads (miRNAs and snoRNAs) can be distinguished from each other and from background RNA in this manner (Fasold et al. 2011; Saçar and Allmer 2014; Abbas et al. 2016; Georgakilas et al. 2020), we investigated whether this remains true when payload-related information is withheld from the classification machinery. To this end, we determined to what extent a random forest classifiers trained on one of the four datasets described in “Datasets” section  is able to distinguish SNHGs, MIRHGs, and NoHGs. The achievable classification accuracy is indicative of the coherence of the RNA classes. We compared classification results based on sequence-only features (in particular weighted *k*-mers), with feature sets extended by (predicted) secondary structures and sequence conservation parameters, respectively (see “Features” section  for further details). The Fickett score was added as extra feature to each comparison, to investigate the influence of coding potential. The robustness of all results is ensured by tenfold cross-validation (10xCV) on all data and feature sets, see Table 2 for a summary.

### Supervised classification of SNHGs, MIRHGs, and NoHGs

*Classification on data that contain the payload* Input data that include the full payload sequences are expected to allow the recognition of the payload type and the absence of a payload, respectively. The classification task on such data therefore also serves as benchmark for our feature-selection strategy. Table 2 summarizes the results for 10xCV on a training/test set split of 80/20. The accuracy of $>\,84\%$ shows that random forests are indeed suitable for the classification task at hand. An interesting finding, however, is that the inclusion of information derived from structure/conservation features leads to a decrease in accuracy, probably due to the high structuredness of both miRNA and snoRNA payloads and similar conservation patterns. We note, finally, that a perfect separation of snoRNAs and miRNA precursors cannot be expected since there is a group of ncRNAs that appear to be in transition between the two functions (Scott et al. 2009; Langenberger et al. 2011; Scott and Ono 2011).

*Classification on sequences flanking the payload* The payload potentially—and in the case of MIRHGs even very likely—contains sequence- or structure-level signals needed for the processing of the payload ncRNA. We therefore have to expect that machine learning techniques will be able to pick up on those signals. To test whether the actual payload of the host genes is necessary, or whether processing-related features in the adjacent sequence are by themselves already sufficient to classify the host-gene type, we restricted *Dataset 2* to cumulatively collected 200 nucleotides of flanking sequence from either side of the payload without overlap and random lncRNA subsequence for NoHGs. We observe that the exclusion of the actual payload sequences had a strong impact on prediction accuracy, which drops from $>\,84\%$ to below $45\%$. For comparison, uniform random sampling would achieve $33\%$ since we consider a balanced three-way classification problem. The integration of structure and conservation features indeed achieves a moderate increase of accuracy to just above $50\%$. The sequences in close vicinity to the actual payload are insufficient to reliably identify the type of payload. Ranking of features according to their importance for classification shows that upon exclusion of the actual payload, sequence and sequence conservation become important features for the classification task (Supplementary Table 2).

*Classification on exons adjacent to the payload* Since snoRNAs are located in introns of the host gene, *Dataset 2* is almost exclusively restricted to intronic regions. However, functions of the matured host genes or other lncRNAs presumably reside in their exonic sequences. Furthermore, sequence and structure features involved in splicing as well as the initial processing of exonic miRNAs are likely to be found in the matured transcript. *Dataset 3*, which is designed to focus on such effects, comprises the 200nt of exonic sequence flanking the payload-bearing intron on each side in the case of an intronic payload or the miRNA precursor in case of exonic miRNAs. Although by far not as good as the accuracy for classification including payloads, we see accuracy rises again to above $66\%$, including structure and conservation features even $>\,70\%$. We conclude that the exonic flanking regions contain more reliable information related to the processing of payload than the flanking sequences in the primary sequence. For miRNAs this can be explained in particular by sequence motifs associated with microprocessor activity (Agranat-Tamir et al. 2014; Pianigiani et al. 2018). For snoRNAs, their obligatory location in (usually short) introns also suggests a connection with splicing, see also Hirose et al. (2003), Kufel and Grzechnik (2019).

*Classification using randomly selected exonic sequences* Based on results from analysis of *Datasets 1–3*, we infer that exonic regions flanking the payload contain some amount of information about the type of the payload. To determine whether this is related to payload itself or a global distinction between the three lncRNAs classes, we constructed *Dataset 4* from random portions of exonic regions excluding the payloads and their immediate vicinity. We reason that these regions are much less likely to contain sequence signals directly related to the processing of the payload than *Dataset 3*. We observe that this is indeed the case in particular if only sequence-level features are included. Although the achievable accuracy on the exonic sequences after inclusion of structure and conservation is higher than for payload flanking sequences of *Dataset 2* ($>\,62\%$ in comparison with $>\,51\%$), it is still well below the accuracy obtained from the flanking exonic regions with $>\,70\%$.

Taken together, our computational experiments indicate that SNHGs, MIRHGs, and NoHGs can be distinguished with reasonable accuracy only based on their payload. Flanking sequence arguably involved in the processing also conveys some pertinent information; however, the achievable classification accuracies are much smaller. If no payload-related sequence information is included, the three lncRNA classes become effectively indistinguishable.

### Influence of specific feature types

In the following we briefly discuss the influence of biological motivated features on the classification tasks. As we shall see, the inclusion of secondary structure and sequence conservation features has a small beneficial effect, but cannot overcome the inability to train an accurate classifier unless payload-related sequence information is included.

*Fickett score* Invented as a measure for coding potential and a go-to feature for separating coding from non-coding RNAs by means of ML (Han et al. 2016), we investigated whether the Fickett score (Fickett 1982) can be used as informative feature for our classification problem as well. While we observed a small increase in accuracy for flanking exons (*Dataset 3*) when only sequence features are used, every other prediction did not profit from its inclusion; thus, the Fickett score does not constitute a meaningful feature for the classification problem at hand.

*RNA secondary structure* SnoRNAs are highly structured RNA species, depending on the correct fold for their biological function. MiRNAs on the other hand are usually loaded into the RISC complex as single strands for prior processing; however, they are also heavily structured. Thus, secondary structuredness, or the lack thereof, could indicate regions which are better suited for the integration of either payload. Using RNAplFold  (Bernhart et al. 2006) of the ViennaRNA  package (Hofacker et al. 1994; Lorenz et al. 2011), we included the probability of being unpaired as feature vectors into our predictions, as explained in detail in “Sequence retrieval and curation” section. In contrast to the Fickett score, inclusion of these features always increased accuracy, although in most cases the improvement is small. In combination with Fickett score, prediction accuracy is even decreased, except for *Dataset 2*, the sequences immediately flanking the payload. Taken together, secondary structure features are informative for the classification task at hand, although their impact appears to be smaller than one might have expected, probably because (conserved) secondary structures are on the one hand abundant across the transcriptome and on the other hand do not seem to be characteristic for lncRNAs (Backofen et al. 2018).

*Sequence conservation* Sequence conservation is a key indicator of biological function. It therefore seems sensible to include conservation features in our classifications. However, this limits application to genomic regions where reliable sequence alignments can be constructed, from which conservation scores can be deducted. We integrated the PhastCons score, i.e., the probability that a given position is part of a conserved region (Siepel and Haussler 2005), as a convenient measure of position-wise conservation, see “Sequence retrieval and curation” section. Similar to structure derived features, adding conservation increases accuracy except when focusing directly on the payload. This effect is strongest when random exonic regions (*Dataset 4*) are used and could hint towards a general trend of conservation differences between the three classes of lncRNAs investigated.

**Table 2:** Overview of tenfold cross-validation accuracy for supervised machine learning on combinations of data and feature sets (*k*-mers only, *k*-mers plus secondary structure, or *k*-mers plus secondary structure and sequence conservation), both with and without the Fickett score as measure of coding potential

Dataset	Fickett score	*k*-mer (%)	+structure (%)	+conservation (%)
Dataset 1	Without	85.50	85.51	84.06
sno: 345, mir: 400, lnc: 400	With	84.06	80.19	82.61
Dataset 2	Without	44.69	47.58	51.69
sno: 690, mir: 800, lnc: 750	With	40.34	43.96	50.72
Dataset 3	Without	66.67	67.03	70.61
sno: 1287, mir: 464, lnc: 2101	With	67.38	65.59	70.25
Dataset 4	Without	42.86	50.00	62.24
sno: 162, mir: 168, lnc: 750	With	41.84	35.71	50.00

### Unsupervised clustering

As an alternative approach to detecting commonalities within the three classes of lncRNAs we considered an unsupervised clustering approach motivated by work of Kirk et al. (2018), who presented a link between *k*-mer profiles and lncRNA function. An initial principal component analysis (PCA) of the four datasets did not reveal any credible clustering or separation between NoHG, MIRHG, and SNHG, see Supplemental Figs. S5–S8. Furthermore, using *k*-means clustering we found that the assignment of the three groups of lncRNAs to the clusters is effectively random: accuracies for all combinations of features and datasets are below $36\%$ (see Supplemental Table S1 and Figs. S1–S4). The use of a convolutional neural network (CNN) with an autoencoder also showed no credible separation of the lncRNA classes. This is not unexpected, however, given the small size of training and test sets. In summary, unsupervised clustering methods appear to be unable to classify lncRNAs by their RNA payload, at least with the data available at present. It appears that the signal that can be obtained from the small miRNAs or snoRNAs is drowned out by the differences among the much larger hosts. This at least suggests that there are no strong features that identify MIRHGs or SNHGs as coherent subgroups in lncRNAs.

### MicroRNA target sites as a feature

At least some SNHGs can act as miRNA sponges (Li et al. 2020; Yang et al. 2019). If this is the primary function of the exonic part of SNHGs, these lncRNAs should be recognizable using the distribution of miRNA binding sites as features for classification. To test this hypothesis, we retrieved predicted and experimentally validated miRNA binding sites from miRTarBase (Huang et al. 2019a), which in contrast to other sources contains not only target sites located in the 3’UTR but also experimentally validated binding site regardless of their genic location. Intersection of this resource with our list of SNHGs and MIRHGs, however, revealed no significant overlap of miRNA binding sites and any of our host lncRNAs. Since less than 0.1% of the reported miRNA target genes are lncRNAs, we used the complete list of seed regions for each miRNA available from TargetScan  (Agarwal et al. 2015) and trained a classifier using only the *k*-mers appearing in these sequences. We restricted our search to regions covered in the analyzed datasets for which we had weighted *k*-mers available. No conclusive enrichment for any of the analyzed seeds could be detected when comparing SNHGs and the other classes. This approach is of course limited due to the rather small regions we cover in our datasets.

## Discussion

In this contribution we asked whether there is a clear distinction between the host genes of snoRNAs and miRNAs on the one hand and lncRNAs without such highly conserved ncRNAs as payloads on the other hand. Our answer is largely negative. While machine learning methods readily distinguish the three classes based on generic features of their respective payloads (or the absence of a miRNA or snoRNA payload, respectively), the classification task appears to become very difficult if the information about the payload and its immediate vicinity is not made available to the classifier. While the sequence adjacent to the payload—in particular in the exonic part of the transcripts—appears to contain some payload-specific information, we found that the association between payload and features obtained from distant regions of the lncRNA is weak. Features derived from structuredness and conservation of these regions have in all cases a positive effect on classification accuracy and are readily ranked among the most important features. They do, however, not fully compensate for the weakness of the association with sequence-based features.

Given that there is mounting evidence for biological function of not only lncRNAs but also specifically for snoRNA and miRNA host genes, we argue that this *lack of detectable association* is of biological interest. It suggests that the function of the host genes is not closely tied to the function of the payload. This is in stark contrast to the protein-coding host genes of snoRNAs, many of which encode ribosomal proteins (Kiss 2002) and thus also contribute to the maturation of the ribosome.

Although a function as miRNA sponge has been reported for many SNHGs, we could not detect features that might connect the sequence or structure of the SNHGs with specific *k*-mers (namely those complementary to the seed regions of miRNAs) or to predicted miRNA target sites. Restricting the data to the small number of experimentally validated miRNA targets only severely limits the power of this feature since only a very small fraction of known target genes are lncRNAs (Karagkouni et al. 2020). The use of predicted target sites, on the other hand, may suffer from high noise levels in the miRNA target predictions. A systematic investigation into miRNA targets on lncRNAs is still missing. Such an endeavor would help to shed light onto the regulatory interplay between SNHGs, MIRHGs, and their payload.

Applying *k*-means clustering to our datasets and features showed poor classification accuracy. Even though lncRNAs as such may harbor sequence motifs that give away conserved RNA binding protein target function among lncRNA groups, our investigation shows only poor classification potential for MIRHGs and SNHGs, even if the payload is considered. No distinctive clusters of MIRHGs and SNHGs were observed. In this respect, our data are consistent with clustering of lncRNA classes observed in Kirk et al. (2018). There, prominent SNHGs such as GAS5 have remained outside the identifiable clusters.

From an evolutionary point of view it may not be surprising that the host genes of miRNAs and snoRNAs do not exhibit recognizable class-specific features. Most likely, the molecular function of the payload, miRNAs, and in particular snoRNAs is much older and pre-dates functions of the non-coding host genes. These most likely arose secondarily, maybe long after the transcripts have come under negative selection as host genes. The lack of common, class-specific features for host genes together with their usually very poor sequence conservation suggests they may even have acquired different functions in different lineages. A better understanding of the host genes thus will require a much more detailed investigation into the patterns of conservation than what is available at present. While the evolution of snoRNAs and miRNAs has been received considerable attention (see, e.g., Berezikov (2011), Hertel and Stadler (2015), Moran et al. (2017), Hoeppner and Poole (2012), Kehr et al. (2014), and the references therein), there are no systematic data on the conservation and evolutionary flexibility of their host lncRNAs.

SNHGs and MIHGs are an excellent system to study the functional evolution of lncRNAs because the conserved payload makes it comparably easy to trace them over much large evolutionary timescales than most other lncRNAs (Nitsche et al. 2015). At the same time, both classes are large enough for statistical and learning-based approaches. With rise of new sequencing technologies and advances in functional screening methods we can expect that more detailed data on host gene functions will be forthcoming. More extensive functional data may also revise the picture of distribution of lncRNA functions, which shows only a rather loose association of biological function and molecular mechanism with sequence and structure features of transcripts.

This does not imply that the host genes do not contribute to the function of the payload. In fact, our data suggest that the sequence flanking miRNAs and snoRNAs indeed contribute to payload processing. Other, more dynamical effects, such as a potential contribution of host gene abundance to the regulation of payload (Baskerville and Bartel 2005; Warner et al. 2018), on the other hand, cannot be assessed at sequence level alone and thus have to remain a topic for future research. Similarly, indirect links between miRNAs and sdRNAs (snoRNA-derived sRNAs) on factors that regulate hostgene transcription as well as direct interactions of the payload with the hostgene, known, *e.g., *in the case of let-7 (Jiao and Slack 2012; Minchington et al. 2020) are beyond the scope of this contribution. Our data do indicate, however, that such feedback will be realized by a diversity of mechanisms, rather than a single ubiquitous paradigm.

## Electronic supplementary material

Below is the link to the electronic supplementary material.

Supplementary material 1 (pdf 368 KB)

Supplementary material 2 (pdf 65 KB)

## Data Availability

All code used in this publication can be found at github (https://github.com:/Bierinformatik/lncRNA_host_gene_classification.git), and data are available from the authors’ Web site http://www.bioinf.uni-leipzig.de/supplements/20-006.
