# Supplementary material for: Are spliced ncRNA host genes distinct classes of lncRNAs?
Source: Theory Biosci. 2020 Nov 21;139(4):349–59. doi: 10.1007/s12064-020-00330-6 (PMC7719101; doi:10.1007/s12064-020-00330-6)
Supplement: Supplementary file 1 — Supplementary material 1 (pdf 368 KB) [file 12064_2020_330_MOESM1_ESM.pdf]

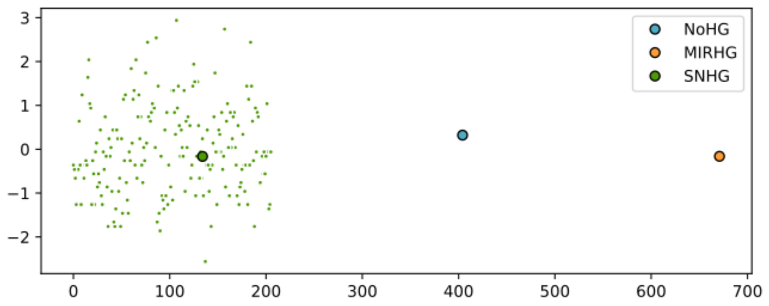

Figure 1. k-means cluster of the test set of dataset 1. All 207 sequences were clustered as SNOHGs (68 MIRHGs, 76 SNOHGs, 63 NoHGs).

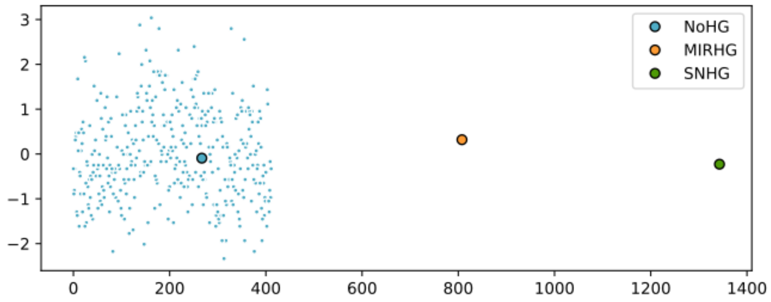

Figure 2. k-means cluster of the test set of dataset 2. All 414 sequences were clustered as NoHGs (127 MIRHGs, 145 SNOHGs, 142 NoHGs).

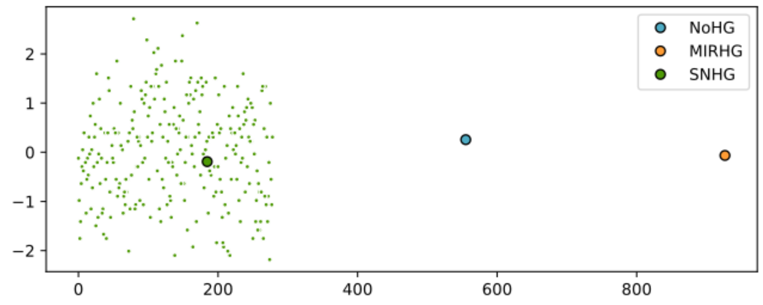

Figure 3. k-means cluster of the test set of dataset 3. All 279 sequences were clustered as SNOHGs (91 MIRHGs, 95 SNOHGs, 93 NoHGs).

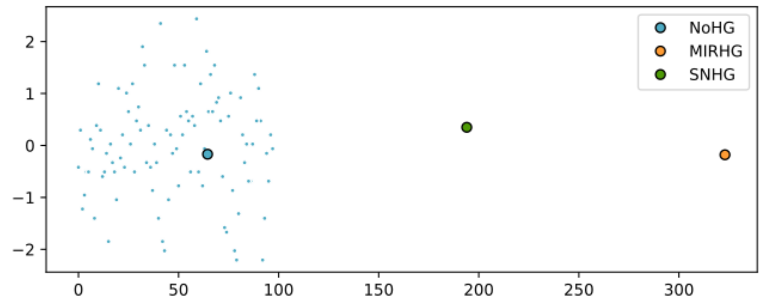

Figure 4. k-means cluster of the test set of dataset 4. All 98 sequences were clustered as NoHGs (26 MIRHGs, 35 SNOHGs, 37 NoHGs.)

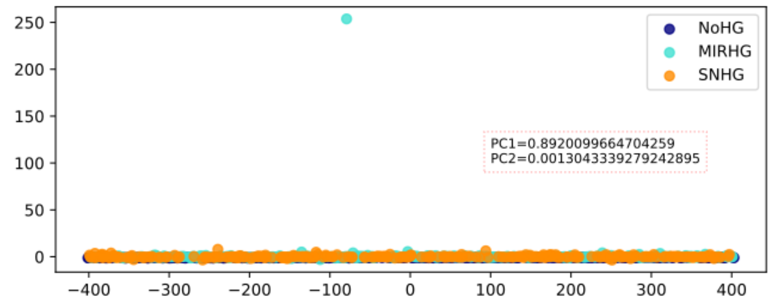

Figure 5. PCA of dataset 1. No separation between datasets is possible.

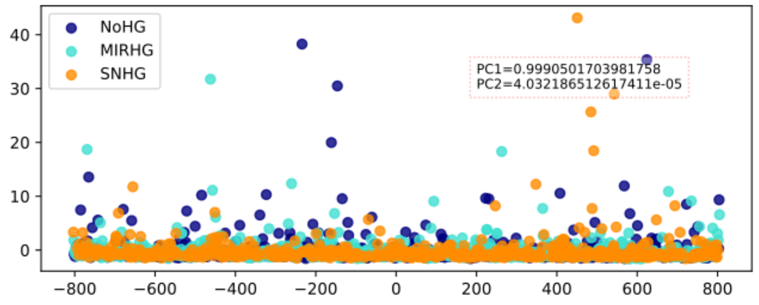

Figure 6. PCA of dataset 2. No separation between datasets is possible.

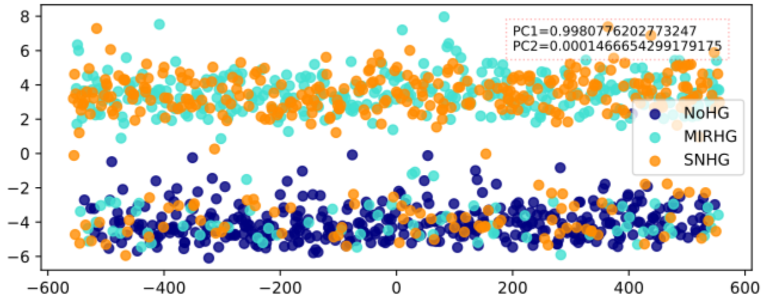

Figure 7. PCA of dataset 3. Although NoHGs cluster, no separation between datasets is possible.

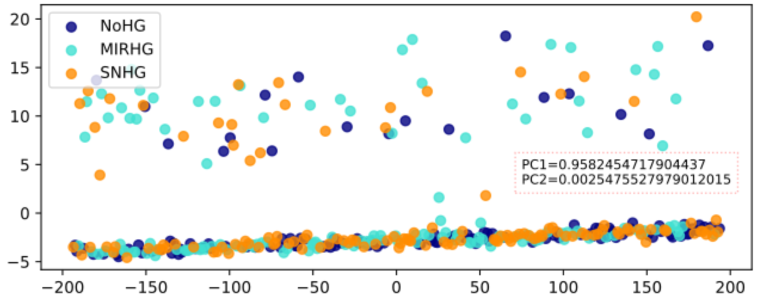

Figure 8. PCA of dataset 4. No separation between datasets is possible.
