# Supplementary material for: Are spliced ncRNA host genes distinct classes of lncRNAs?
Source: Theory Biosci. 2020 Nov 21;139(4):349–59. doi: 10.1007/s12064-020-00330-6 (PMC7719101; doi:10.1007/s12064-020-00330-6)
Supplement: Supplementary file 2 — Supplementary material 2 (pdf 65 KB) [file 12064_2020_330_MOESM2_ESM.pdf]

Table 1. Supplemental Table S1: Results for k-means clustering. The *Training Accuracy* column shows the accuracy on the training set, with the count of accurately predicted labels in the *Correctly predicted* column. We show the frequencies of each class in both the training set and the test set in the top row in the following two columns, respectively, with the actual predicted labels in the bottom row. The datasets were split 80-20.

	Training Accuracy	Correctly predicted (Total)	Labels in trainingset (lnc, mir, sno)<br>Predicted labels [lnc, mir, sno]	Labels in testset (lnc, mir, sno)<br>Predicted labels [lnc, mir, sno]
Dataset 1	34.42%	285 (828)	(282, 277, 269)<br>[278,272,278]	(63, 68, 76)<br>[0, 0, 207]
Dataset 2	33.33%	579 (1656)	(548, 563, 545)<br>[549,557,550]	(142,127,145)<br>[414, 0, 0]
Dataset 3	35.76%	398 (1113)	(371, 373, 369)<br>[371,372,370]	(93, 91, 95)<br>[0, 0, 279]
Dataset 4	34.79%	135 (388)	(125, 136, 127)<br>[130, 129, 129]	(37, 26, 35)<br>[98, 0, 0]

Table 2. Supplemental Table S2: Five most important features for classification according to the random forest classifier.. *x-zmers* should be interpreted as all kmers from length x through z.

Dataset	Featureset	Fickett Score	Rank 1	Rank 2	Rank 3	Rank 4	Rank 5
Dataset 1	kmer	without	(3-4mers)	(CG, 3-5mers)	(3-4mers)	(3-6mers)	(3-5mers)
		with	(2-4mers)	(2mers, 4-5mers)	(3-4mers)	(3-4mers)	(4-5mers)
	+structure	without	(4-5mers)	(4-5mers)	(4-5mers)	(4-5mers)	(4mers)
		with	(3-4mers)	(4mers)	(4-5mers)	(4-6mers)	(4-6mers)
	+conservation	without	(CCG, 4-6mers)	(3-4mers, AGCGG)	(CG, 3-5mers)	(CG, 3-7mers)	
		with	(3-4mers, AGCGG)	(CG, 3-7mers)	(CGA, 4-5mers, 7mers)	(3-4mers, CAGCG)	(CG, 3-7mers)
Dataset 2	kmer	without	GC content	Sequence	(2mers)	(2mers)	(2mers)
		with	Sequence	GC content	Fickett score	(2mers)	(2mers)
	+structure	without	GC content	Structure	Structure	Sequence	Structure
		with	GC content	Structure	Fickett score	Structure	Structure
	+conservation	without	Conservation	GC content	Sequence	Conservation	Structure
		with	Conservation	GC content	Conservation	Structure	Sequence
Dataset 3	kmer	without	GC content	(7mers)	Sequence	(3-4mers)	(3-4mers)
		with	GC content	(7mers)	Fickett score	Sequence	(7mers)
	+structure	without	GC content	(7mers)	Structure	Structure	Structure
		with	GC content	(7mers)	(7mers)	Structure	Structure
	+conservation	without	GC content	(7mers)	(3-4mers)	Structure	Structure
		with	(7mers)	GC content	Structure	(7mers)	Structure
Dataset 4	kmer	without	GC content	Sequence	(2mers)	(2mers)	(2-3mers, AAAAA, AAAAAA)
		with	GC content	Fickett score	Sequence	(2mers)	(2-3mers, AAAC, 5-6mers)
	+structure	without	GC content	Structure	Structure	Structure	Structure
		with	Structure	GC content	Structure	Structure	Sequence
	+conservation	without	GC content	Structure	Sequence	Structure	Structure
		with	GC content	Structure	Structure	Structure	Structure

Table 3. Accuracy after hyperparameter tuning by grid search using 10-fold cross validation metric for supervised machine learning. Indicated are accuracy per feature set and parameters for each feature set as [criterion, number of trees]. Bootstrap and out-of-bag score was always enabled.

Dataset	Fickett Score	kmer		+structure		+conservation	
Dataset 1	without	85.50%	['gini', 300]	85.51%	['entropy', 100]	84.06%	['entropy', 1000]
SNHGs: 345, MIRHGs: 400, NoHGs: 400	with	84.06%	['gini', 500]	80.19%	['gini', 500]	82.61%	['entropy', 100]
Dataset 2	without	44.69%	['entropy', 1000]	47.58%	['entropy', 1000]	51.69%	['entropy', 100]
SNHGs: 690, MIRHGs: 800, NoHGs: 750	with	40.34%	['entropy', 300]	43.96%	['entropy', 500]	50.72%	['entropy', 1000]
Dataset 3	without	66.67%	['gini', 1000]	67.03%	['entropy', 500]	70.61%	['gini', 100]
SNHGs: 1287, MIRHGs: 464, NoHGs: 2101	with	67.38%	['entropy', 100]	65.59%	['entropy', 500]	70.25%	['gini', 300]
Dataset 4	without	42.86%	['entropy', 1000]	50.00%	['gini', 100]	62.24%	['gini', 500]
SNHGs: 162, MIRHGs: 168, NoHGs: 750	with	41.84%	['entropy', 1000]	35.71%	['entropy', 100]	50.00%	['entropy', 100]
